# *In Vitro* Testing of Biomaterials for Neural Repair: Focus on Cellular Systems and High-Content Analysis

**DOI:** 10.1089/biores.2016.0025

**Published:** 2016-08-01

**Authors:** Vito Antonio Baldassarro, Luisa Stella Dolci, Chiara Mangano, Luciana Giardino, Chiara Gualandi, Maria Letizia Focarete, Laura Calzà

**Affiliations:** ^1^ Health Sciences and Technologies—Interdepartmental Center for Industrial Research (HST-ICIR), University of Bologna, Bologna, Italy.; ^2^ Department of Pharmacy and Biotechnology (FaBit), University of Bologna, Bologna, Italy.; ^3^ Department of Veterinary Medical Science, University of Bologna, Bologna, Italy.; ^4^ Department of Chemistry “G. Ciamician” and National Consortium of Materials, Science, and Technology (INSTM, Bologna RU), University of Bologna, Bologna, Italy.

**Keywords:** Good Laboratory Practice guidelines, neural cell lines, neural primary culture, neural stem cells high-content analysis, poly(lactic acid)

## Abstract

Biomimetic materials are designed to stimulate specific cellular responses at the molecular level. To improve the soundness of *in vitro* testing of the biological impact of new materials, appropriate cell systems and technologies must be standardized also taking regulatory issues into consideration. In this study, the biological and molecular effects of different scaffolds on three neural systems, that is, the neural cell line SH-SY5Y, primary cortical neurons, and neural stem cells, were compared. The effect of poly(L-lactic acid) scaffolds having different surface geometry (conventional two-dimensional seeding flat surface, random or aligned fibers as semi3D structure) and chemical functionalization (laminin or ECM extract) were studied. The endpoints were defined for efficacy (i.e., neural differentiation and neurite elongation) and for safety (i.e., cell death/survival) using high-content analysis. It is demonstrated that (i) the definition of the biological properties of biomaterials is profoundly influenced by the test system used; (ii) the definition of the *in vitro* safety profile of biomaterials for neural repair is also influenced by the test system; (iii) cell-based high-content screening may well be successfully used to characterize both the efficacy and safety of novel biomaterials, thus speeding up and improving the soundness of this critical step in material science having medical applications.

## Introduction

The potential applications of tissue engineering products for regenerative medicine in acute and chronic diseases of the nervous system are quite wide. There is an extensive literature aimed to compare the biological impact of different scaffolds for neural repair, considering the chemical composition of the materials, architecture, functionalization, etc.^1^ However, these studies often brought to contradictory results when translated in animals, and this could be at least partially due to the different cell systems and readout technologies used.^2^ In fact, key biological differences in the cellular test systems (i.e., cellular composition, maturation time, culture media composition, growth factor production, etc.) make it very hard to compare results and judge the “biomaterial effect on neural cells”.^3,4^

Therefore, in this work we compared the biological and molecular effects of different materials on three cell systems commonly used for these purposes, that is, the neural cell line SH-SY5Y, primary cortical neurons, and neural stem/precursor cells (NSC). We investigated glass substrates and poly(L-lactic acid) (PLLA) scaffolds having different surface geometry (conventional two-dimensional seeding [2D] flat surface, random or aligned fibers as semi3D structure) and chemical functionalization (laminin or ECM extract).

We investigated defined endpoints for efficacy (i.e., neural differentiation and neurite elongation) and safety (i.e., cell death/survival). We also introduced high-content analysis compared with conventional biochemical and morphological readout methods, and the international guidelines for the “mutual acceptance of data” for safety/toxicology tests (Organization for Economic Co-operation and Development–OECD-).^5,6^

## Materials and Methods

Detailed methods are provided in the Supplementary Data, Materials and Methods section.

### Materials

PLLA (Lacea H.100-E) (M_w_ = 8.4 × 10^4^ g/mol, PDI = 1.7) was supplied by Mitsui Fine Chemicals. Dichloromethane (DCM), dimethylformamide, dimethyl sulfoxide, bovine serum albumin, laminin 1 mg/mL, Triton, paraformaldehyde, retinoic acid (RA), penicillin, and streptomycin were purchased from Sigma-Aldrich and were used without any further purification. Hoechst 33258, Cultrex^®^ basement membrane extract (Trevigen^®^), DMEM-F12, 1X Minimum Essential Medium/Non Essential Aminoacids, and 1X Penicillin–Streptomycin purchased from Gibco-Invitrogen, fetal bovine serum (Euroclone), and NaOH 10 mM (Carlo Erba) were used.

### Cell substrates fabrication, characterization, and chemical functionalization

PLLA films were prepared by the solvent cast method, from a 10% w/v polymer solution in DCM at room temperature. Electrospun scaffolds were fabricated by using a commercial electrospinning machine equipped with a temperature and humidity control system (Spinbow S.r.l.). The electrospun scaffolds were characterized by means of scanning electron microscopy (SEM) and static water contact angle (WCA) measurements.

Electrospun PLLA random and aligned scaffolds, and PLLA-film were cut and assembled by means of CellCrown supports (Scaffdex) in a 24-well. Cover glasses and electrospun were sterilized and functionalized with laminin and Cultrex BME.

### Cell cultures

Human neuroblastoma SH-SY5Y cells were differentiated on different surfaces (Cultrex-coated glass, laminin-coated glass, laminin-coated or not PLLA-FILM, laminin-coated or not random and aligned electrospun PLLA scaffold) using 10 μM RA and cultured for 7 days.

Primary neurons were isolated from newborn mice cortex and cytosine-arabinofuranoside (10 μM; Sigma) was added to the culture medium, to inhibit proliferation of the glial cells,^7^ and cultured for 15 days.

NSCs were obtained from embryos forebrain and secondary neurospheres were dissociated and plated at a density of 1 × 10^4^ cells/cm^2^ in the same culture medium without mitogens.^8,9^ To achieve full lineage commitment and cell differentiation, seeded cells were cultured for 15 days.

Animal care and treatment were in accordance with the EU Directive 2010/63/EU and approved by the Ethics Committee of Animal Experimentation, University of Bologna.

### Viability assays

Cell viability was determined by high-content analysis (HCS) as the percentage of pyknotic nuclei calculated by Cellomics^®^ Compartmental Analysis BioApplication, using “population characterization” parameter. MTT assay was performed on primary cortical neurons seeded on 2D Cultrex-coated surface and semi3D-aligned uncoated scaffolds. Absorbance was measured at 570 nm.

### Immunocytochemistry

Cells were fixed with 4% paraformaldehyde. Cell-specific proteins were identified by indirect immunofluorescence using mouse anti-β-III-tubulin (1:2000; R&D Systems); rabbit anti-GFAP (1:500; Dako) and rabbit anti-MBP (1:250; Dako) antisera. The anti-rabbit DyLight 568- and anti-mouse AlexaFluor 488-labeled (1:500; Invitrogen) were used as secondary antisera. For nuclear staining, cells were incubated with 1 μg/mL Hoechst 33258.

### Fluorescence microscopy, image analysis, and HCS

Fluorescence images were taken with 10X objective (10X/0.45 N.A. Plan Fluor Nikon) using NIKON Eclipse E600 (Nikon) equipped with digital CCD camera QImaging Retiga 20002V (QImaging), and analyzed using Imaging NIS Elements software (Nikon). To analyze neurites outgrowth of SH-SY5Y cells, the length of individual neurites in each acquired images was measured. Each sample was repeated twice and data were expressed as mean length ± SEM.

HCS was performed using CellInsight NXT (Thermo Scientific) equipped with digital CCD camera Photometric X1. All images were taken with 10X objective (10X/0.30 N.A. Plan Fluor Olympus) using 4 × 4 binning (552 × 552) and analyzed using Cellomics Scan Version 6.4.4 (Thermo Scientific). For each well examined, multiple fields were sampled. Within each field, matched fluorescent images of Hoechst-labeled nuclei, β-III-tubulin/AlexaFluor 488-labeled cells were acquired using 386/23 (Channel1), 485/20 (Channel2) filters, respectively. Neurite analysis was performed with Cellomics Neuronal Profiling BioApplication. Identification of nuclei, cell bodies, and neurites was optimized using 10 representative images for each condition tested.

Lineage analysis was performed by Cellomics Compartmental Analysis BioApplication, using “population characterization.” After the nuclei identification, the user chooses the area around the nucleus and the fluorescence intensity threshold.

### Statistical analysis

The study design included at least three independent experiments and data reported in graphs are the mean ± standard deviation (SD) of the different experiments. Statistical testing was performed with ANOVA followed by appropriate *post-hoc* multiple comparisons test (see legend to the figures for details). Student's *t*-test was also used. A probability level of *p* < 0.05 was considered to be statistically significant.

## Results

The following variables were included in the study: (A) *materials and topography of cell substrates*: conventional glass/plastic 2D, semi three-dimensional random electrospun PLLA scaffolds (semi3D, random) and semi three- dimensional aligned electrospun PLLA scaffolds (semi3D, aligned); (B) *substrate chemical functionalization*: no-coating, laminin coating of the above materials and whole ECM extract coating of the above materials; (C) *neural cell type*: SH-SY5Y neural differentiated cell line, primary cortical neurons, and neural stem cells; (D) *assay and technologies for toxicology testing*: conventional low-throughput and cell-based high throughput screening.

### Scaffold fabrication and chemical functionalization

**FIG. 1.**
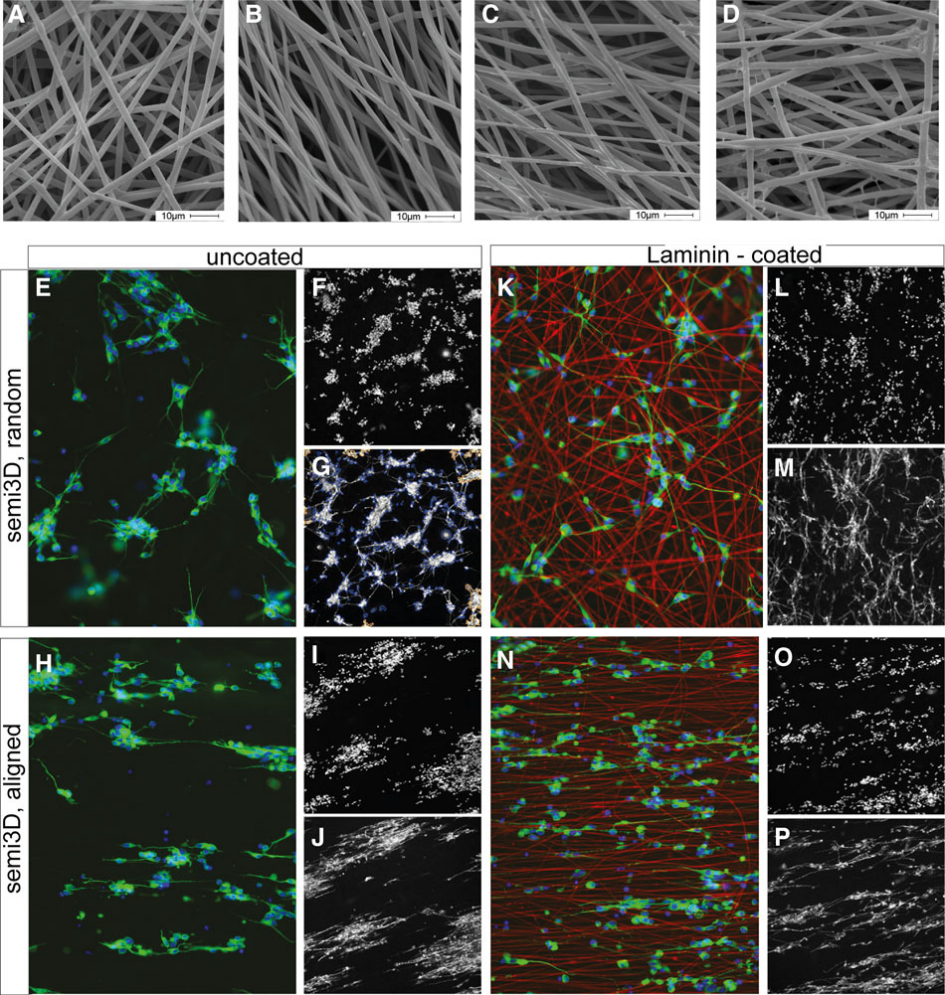
Scaffold characterization: morphology and cell seeding. SEM micrographs of **(A)** semi3D-random scaffold; **(B)** semi3D-aligned scaffold; **(C)** semi3D-random scaffold coated with laminin; **(D)** semi3D-random scaffold coated with Cultrex. Scale bar: 10 μm. **(E–P)** Conventional fluorescence micrographs showing the SH-SY5Y cell deposition (labeled by β-III-tubulin) on the semi3D uncoated **(E–J)** and laminin-coated **(K–P**; laminin is in red**)** PLLA scaffolds, having random **(E–G; K–M)** and aligned **(H–J; N–P)** topography **(E, H, K, N)**; HCS visualization of the same cell preparations (semi3D, uncoated, random: **F,G**; semi3D, uncoated, aligned: **I,J**; semi3D, laminin-coated, random: **L,M**; semi3D, laminin-coated, aligned: **O,P**). HCS, high-content analysis; PLLA, poly(L-lactic acid); SEM, scanning electron microscopy.

PLLA films and electrospun fibrous scaffolds were fabricated to study the influence of substrate topography on neuron viability and differentiation. Scaffolds made of random and aligned fibers were compared (Fig. 1A,B). PLLA fibers without bead defects were obtained with comparable micrometric mean diameters (semi3D-random: 1.8 ± 0.4 μm; semi3D-aligned: 1.8 ± 0.6 μm). To improve wettability and, thus, biocompatibility of the polymer, electrospun scaffolds were coated with ECM proteins, that is, laminin (Fig. 1C), and the ECM extract Cultrex containing laminin, collagen IV, entactin, and heparin sulfate proteoglycan (Fig. 1D).

In both cases, the morphology of the coated fibers did not appear to be significantly altered by the ECM proteins, and the coating was found to cover quite homogeneously the fiber surface (Fig. 1K,N), without modifying the porous structure of the scaffold.

**FIG. 2.**
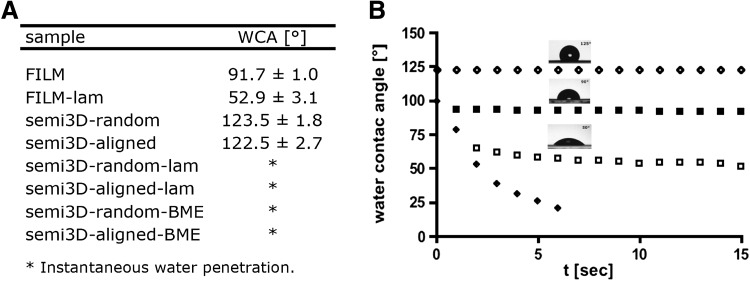
Scaffold characterization: wettability. **(A)** mean WCA values; **(B)** representative WCA behavior for semi3D-random scaffold (white diamonds), semi3D-random scaffold laminin coated (black diamonds), PLLA-FILM (black squares) and PLLA-FILM laminin coated (white squares); representative images of the water drop are also reported. WCA, water contact angle.

To investigate the surface hydrophilicity of the scaffolds, WCA measurements were performed and results are shown in Figure 2A,B, where the mean WCA values are reported for all investigated samples, together with the representative behavior of the WCA versus time for selected samples and the water drop images corresponding to a highly hydrophobic (top), intermediate (middle), and hydrophilic (bottom) scaffold. As expected, the semi3D-random and the semi3D-aligned electrospun scaffolds show a similar hydrophobic behavior with a WCA higher than that of the corresponding PLLA film (Fig. 2A), due to their high porosity and air entrapment into pores. Laminin and ECM treatment dramatically lowered the WCA of the electrospun scaffolds (Fig. 2A,B), whereas the laminin coating of the PLLA film increased only slightly the hydrophilicity (Fig. 2A). In the semi3D-laminin-coated scaffold and semi3D-Cultrex-coated scaffold the water drop was spread out instantaneously and immediately penetrated into the scaffold, resulting in a change of the WCA from about 120° to about 20° (or lower) in less than 10 sec.

Figure 1E–P illustrates cell deposition (SH-SY5Y) on the semi3D-uncoated (E–J) and laminin-coated (K–P) PLLA scaffolds, having random (E–G; H–J) and aligned (K–M; N–P) topography, as visualized by conventional microscopy (E, H, K, N) and HCS (F, G, I, J, L, M, O, P).

### Scaffolds and neural differentiation

The biological properties of the three different neural systems included in the study were compared on the different 2D/ semi3D substrates and functionalization, in the same experimental assay and using high-throughput analysis. Neurite elongation was taken as primary endpoint for morphological maturation using the cytoskeleton marker β-III-tubulin.

**FIG. 3.**
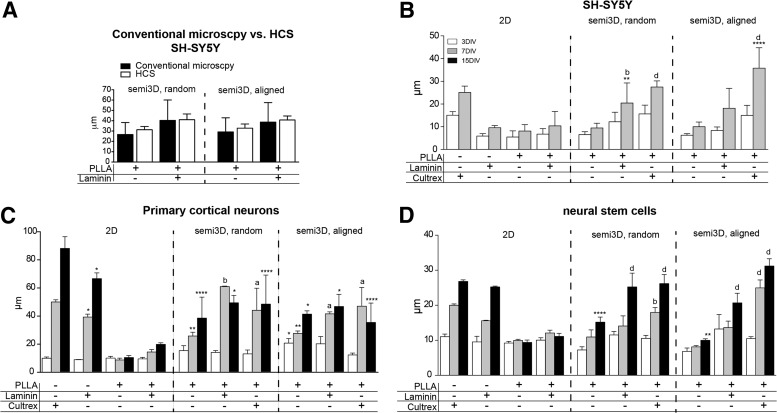
Effect of different scaffolds on neural differentiation. **(A)** Neurite length of SH-SY5Y cells grown on different scaffolds, as measured by conventional quantitative microscopy and HCS. **(B)** Neurite length of SH-SY5Y cell line, as measured by HCS; **(C)** Neurite length of primary cortical neurons, as measured by HCS; **(D)** Neurite length of neurons derived from NSCs, as measured by HCS. In all cell systems, conventional 2D, semi3D-random and semi3D-aligned scaffolds having different coatings are compared. Data are expressed as mean ± SD, and represent the mean of three independent experiments. Statistical analysis: two-way ANOVA (SH-SY5Y F[9, 100] = 5.165; primary cortical neurons F[14,45] = 6.317; NSCs F[18,60] = 15.40) followed by Tukey's *post hoc* test. Asterisks represent the difference between the semi3D group versus the relative 2D control group within the same DIV (*p < 0.05; **p < 0.01; ***p < 0.001; ****p < 0.0001), letters indicate the difference between coated semi3D groups versus the relative uncoated semi3D control group (a = *p* < 0.05; b = *p* < 0.01; d = *p* < 0.0001). 2D, two-dimensional seeding; SD, standard deviation.

The use of HCS automatic procedure versus conventional computerized morphometry was first validated on semi3D cell systems using SH-SY5Y grown on laminin-coated and uncoated random electrospun scaffolds (Fig. 3A). For conventional morphometry, at least nine different fields were taken to obtain a minimum number of 300 neurites (200 cells), whereas for HCS analysis all the cells (a mean of 100,000 cells) were analyzed. The mean length of neurites obtained with the two techniques was equivalent and, as expected in view of the larger number of cells analyzed, the SD derived from HCS analysis was four times smaller compared with conventional morphometry.

We then compared the neurite elongation of the three different neural cells by using HCS. Results are presented in Figure 3, where panel B refers to SH-SY5Y, panel C to primary neurons, and panel D to neural stem cells. In each graph, results are grouped according to the topographic conditions (2D, semi3D random, semi3D aligned), chemical functionalization (PLLA alone, laminin, Cultrex, as indicated in the graphs), and maturation times (3, 7, 15DIV). The statistical analysis is reported in the figure legend.

In all topographic conditions PLLA alone impairs neurite elongation in all cell systems, whereas chemical functionalization with ECM extract favors neurite elongation of both SH-SY5Y and NCSs, an effect that is maximized by alignment. Conversely and regardless of the chemical coating of the PLLA scaffold, semi3D topography impairs maturation of primary cortical neurons. Actually, while SH-SY5Y cells attach to 2D PLLA and then survive, primary neurons and NSC do not attach to 2D PLLA. Notably, semi3D topography seems to overcome this problem even in the absence of chemical coating with ECM extracts or proteins. Moreover, aligned PLLA clearly directs the neurite elongation in a fashion parallel to the fibers.

### Scaffolds and glial/neuronal lineage

**FIG. 4.**
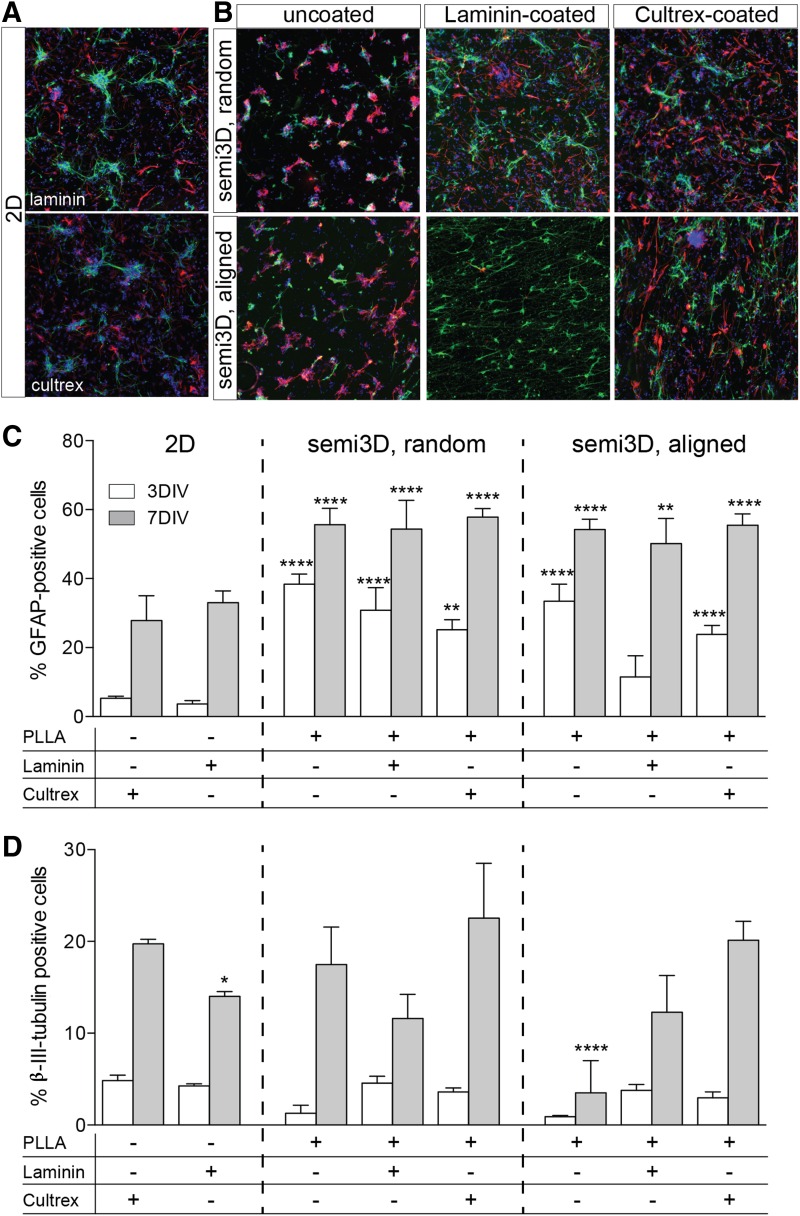
Effect of different scaffolds in the neural and astroglial specification of NSCs. **(A, B)** Representative images of plated NSCs at 7DIV grown on 2D **(A)** and semi3D scaffolds **(B)**, stained with β-III-tubulin (green), GFAP (red) and Hoechst (blue). **(C, D)** The graphs indicate the percentage of GFAP **(C)** and β-III-tubulin **(D)**-positive cell, according to the PLLA topography and coating. Data are expressed as mean ± SD, and represent the mean of three independent experiments. Statistical analysis: two-way ANOVA (GFAP F[7, 32] = 3.404; β-III-tubulin F[7, 30] = 7.136) followed by Tukey's post test. Asterisks represent the difference between the semi3D group versus the relative 2D control group within the same DIV (*p < 0.05; **p < 0.01; ***p < 0.001; ****p < 0.0001).

**FIG. 5.**
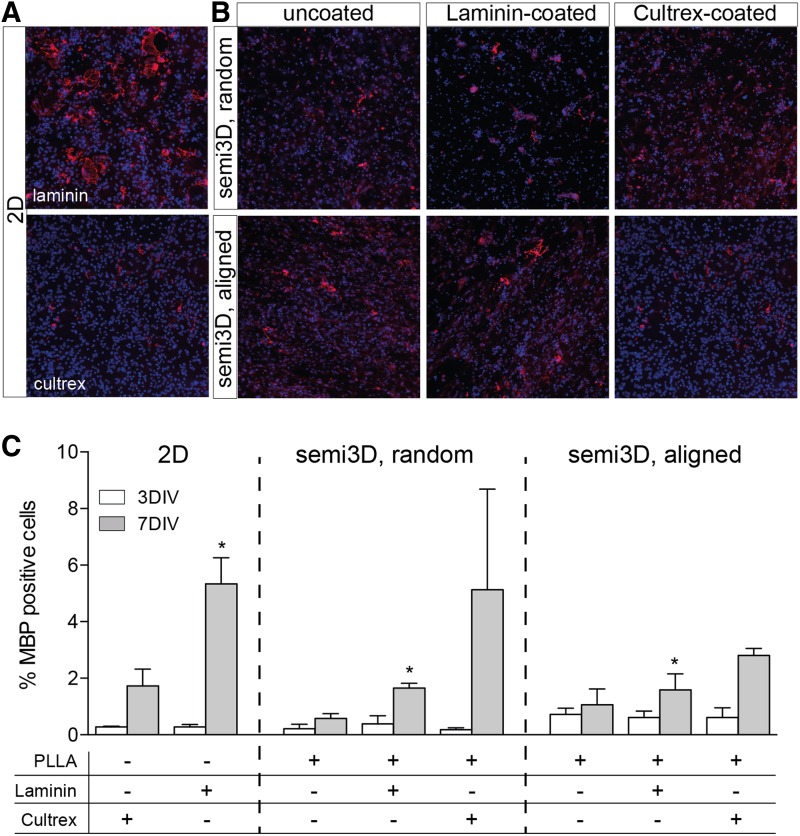
Effect of different scaffolds on oligodendroglial specification of NSCs. **(A, B)** Representative images of plated NSCs at 7DIV grown on 2D **(A)** and semi3D scaffolds **(B)**, stained with MBP (red) and Hoechst (blue). **(C)** The graph represents the percentage of MBP positive cells. Data are expressed as mean ± SD, and represent the mean of three independent experiments. Statistical analysis: two-way ANOVA (F[7, 28] = 4.739) followed by Tukey's post test. Asterisks represent the difference between the semi3D group versus the relative 2D control group within the same DIV (*p < 0.05; **p < 0.01; ***p < 0.001; ****p < 0.0001).

The capability of PLLA to influence cell lineage in primary cultures (Supplementary Fig. S1) and NSCs (Fig. 4) was then investigated, both as a chemical entity and as regards the topographic arrangement. In both cell systems, the semi3D PLLA arrangement strongly increases the percentage of astrocyte (GFAP-positive cells) in the culture, both at early (3DIV) and late (7DIV) time in culture. Chemical functionalization does not substantially modify the astroglial fate from NSC, whereas it decreases the PLLA effect in primary neurons. In the same experimental conditions, also the oligodendroglial lineage and maturation was analyzed by evaluating the percentage of cells expressing MBP, which is a protein expressed in terminally differentiated oligodendrocytes (Fig. 5). First of all, it was observed that the PLLA coating of 2D substrate completely prevents adhesion of oligodendrocyte precursors, and so no mature cells are observed. Semi3D topography allows a better adhesion, and Cultrex coating maximizes the effect of topography. However, it should be mentioned that the culture conditions were optimized for neural differentiation, and so chemical agents known to improve oligodendroglial lineage and maturation were not included in the culture medium.

### Effect of scaffolds on cell viability

**FIG. 6.**
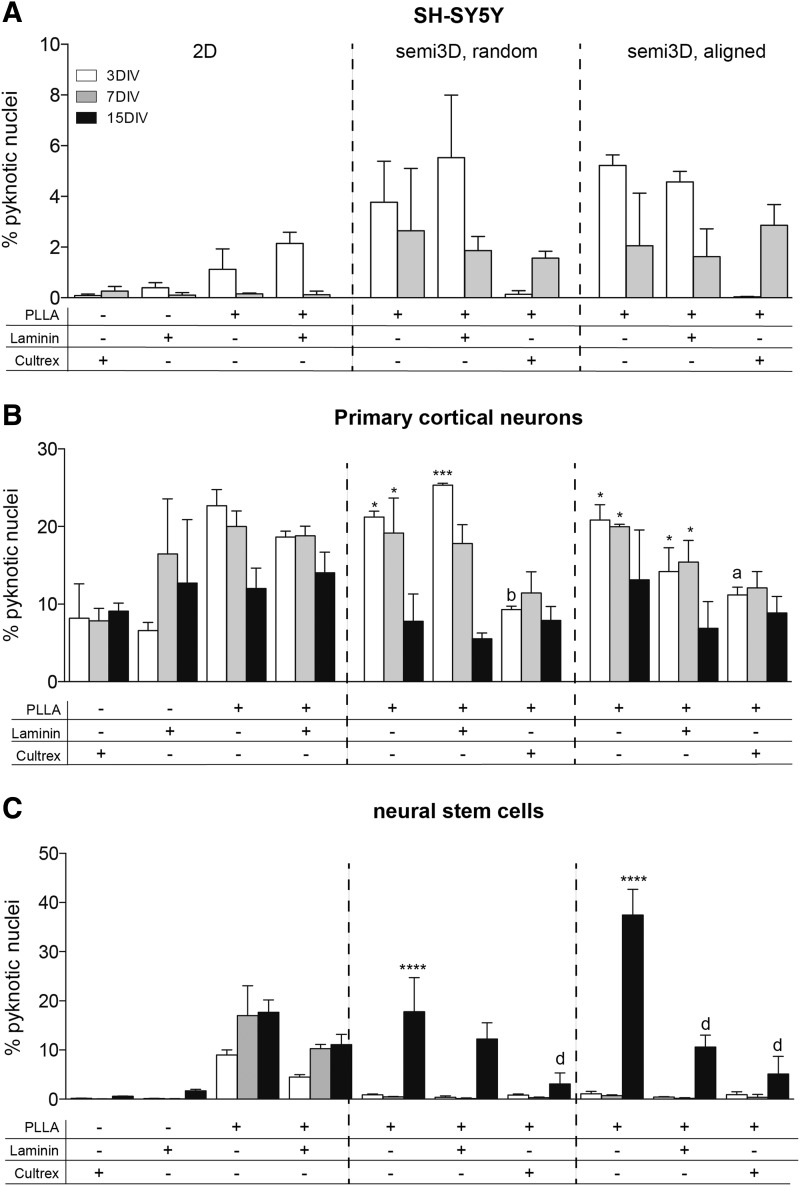
Effect of different scaffolds on cell viability of SH-SY5H cell line **(A)**, primary cortical neurons **(B),** and NSCs **(C)**. The graphs represent the percentage of pyknotic nuclei measured using HCS technology. Data are expressed as mean ± SD, and represent the mean of three independent experiments. Statistical analysis: two-way ANOVA (*n* = 3; SH-SY5Y F[9, 60] = 5.682; primary cortical neurons F[14, 40] = 4.164; NSCs F[18, 60] = 23.57) followed by Tukey's post test. Asterisks represent the difference between the semi3D group versus the relative 2D control group within the same DIV (*p < 0.05; ***p < 0.001; ****p < 0.0001), letters indicate the difference between coated semi3D groups versus the relative uncoated semi3D control group (a = *p* < 0.05; b = *p* < 0.01; d = *p* < 0.0001).

Safety and toxicology testing is a critical step in the biomaterials development as required by the regulatory agencies. We first evaluated the possibility of applying HCS to semi3D cell systems to determine cell viability compared with conventional biochemical methods. Both MTT and HCS-based nuclear morphology indicate that the semi3D-aligned fibers strongly reduce cell survival (Supplementary Fig. S2). HCS was then used to study the toxicity of materials on SH-SY5Y, primary neurons, and neural stem cells, by evaluating the percentage of pyknotic nuclei over all seeded cells. Results are presented in Figure 6, grouped according to the effect of different scaffolds and substrates on SH-SY5Y (A), primary cortical neurons (B), and neural stem/progenitor cells (C). A mean of 300,000 cells/wells, three wells/condition were included in the analysis (mean cell number in each analysis was 900,000 cells/group). In 2D conventional culture conditions, cells from the different systems display substantial differences in viability. Viability of SH-SY5Y cells is not affected by any of the investigated experimental conditions (Fig. 6A), whereas primary cortical neurons show a higher spontaneous cell death even when grown in ideal culture conditions (Cultrex) (percentage of pyknotic nuclei on 2D Cultrex-coated glass: SH-SY5Y 0.26 ± 0.07; primary cortical neurons 9.10 ± 0.59; *p* < 0.0001). The impact of semi3D topography on cell viability was then examined by comparing the semi3D (e.g., PLLA scaffolds) versus the relative 2D (e.g., conventional glass/plastic seeding) control group at the same DIV (asterisks), whereas the effect of the coating was analyzed by comparing the semi3D-coated versus the related uncoated semi3D control group (letters), by one-way ANOVA followed by the Tukey's test. When using NSCs as cell system, a dramatic effect of semi3D PLLA scaffold on cell viability is observed in fully differentiated cells, when PLLA random and aligned fiber distribution causes 20% and 40% cell death, respectively (Fig. 6C). Notably, both laminin and Cultrex functionalization protects cell viability almost completely.

## Discussion

In this study, we showed that the effect of biomaterials on both neuron viability and differentiation is strongly influenced by the cellular system used for the test. We also showed that cell-based HCS technology can be applied to biomaterial *in vitro* testing, thus improving robustness and statistical consistency of the results.

The right cell-culture has actually been recognized as one of the six most important technical determinants of project success and pipeline quality by the pharmaceutical industry.^10^ We then tested the effect of PLLA scaffold on viability and differentiation using three cell systems widely used in the field, that is, the SH-SY5Y cell line, cortical primary neurons, and neural stem cells. The SH-SY5Y neuroblastoma cell line is derived from human tissue, can be easily differentiated toward a neuronal-like cell line, and is a widely available, easy-to-handle, and highly reproducible system.^11^ However, its sensitivity to a wide range of toxic stimuli is lower compared with primary neurons,^12^ and neurite outgrowth may be different from that occurring in primary neurons.^13,14^ Primary neurons and neural stem cells are more physiologically significant and the possibility of false positives and negatives is smaller compared with cell lines, thus meeting the basic requirement for *in vitro* safety testing, that is, to represent the *in vivo* situation as closely as possible. However, both require selective skills for handling and result interpretation.^15,16^

The most relevant result from this study is the different safety profiles of the same biomaterial obtained using different neural systems. While viability of SH-SY5Y cells is not modified by the substrates, either primary neurons and neurons derived from stem cell showed a very high degree of mortality on PLLA, that is almost completely prevented by chemical coating with ECM proteins. This result also has implications to establish the appropriate guidelines to test the safety-profile of the materials and fabrication for regulatory decision makers.^3,17^

On the contrary, the effect of PLLA scaffold on differentiation, as established by neurite elongation and branching, is quite similar in all neural cell types. In particular, it was demonstrated that all three cell systems are negatively affected by the PLLA uncoated scaffolds in terms of neurite length, but that ECM protein coating differentially affects the neuron maturation process. Cultrex coating strongly favors neurite elongation of SH-SY5Y cells on semi3D scaffolds, whereas semi3D surfaces, whether coated or not, negatively affect the neurite elongation of primary cortical neurons. Conversely, PLLA scaffolds impair the neurite elongation of neurons derived from NSCs, but this effect is exceeded by Cultrex coating. As already reported,^18–20^ aligned fibers display the best ability to guide cell orientation. Our results confirm that the incorporation of ECM molecules favors biological processes, such as cell adhesion, neurite elongation, and cell differentiation.^21–27^

However, the different cell compositions in primary cultures and NSC might also be considered. Primary neurons and NSC-derived neurons contain a different percentage of astrocytes, also according to the chemistry and topography of the substrate. In the same conditions in which we observed an increased percentage of astroglial fate from NSC, also an increase in neurite length was observed, thus raising the question of whether this is because of a direct effect of the topography and chemistry of the culture substrate, or to an indirect effect due to the supporting action of astrocytes on neurons. Astroglial cells actually attach more strongly to microfabricated pillars than to smooth substrates,^28^ and the presence of more astrocytes in the test system could itself affect neuronal maturation, neurite elongation, and branching.

## Conclusions

Improving the standards for basic and preclinical research is a recognized need to overcome the weakness that pervades the current system of basic and preclinical research, and to increase reproducibility in science.^4,29–31^ The statistical consistency of the *in vitro* data could be improved by the extensive use of high-throughput techniques compared with low-throughput conventional analysis. Cell-based high-throughput technology combining cellular imaging with high-throughput data analysis,^14^ is successfully applied to drug screening,^32,33^ and it was also used to set up standard procedures according to the ECVAM Good Cellular Culture Practice guidelines.^34^ In this study we used HCS, combining in the same assay the safety profile (i.e., nuclear morphology) with the efficacy profile (i.e., neurite elongation). We confirmed that HCS is a quite robust technological platform for *in vitro* testing for materials^35^ that can truly improve the pipeline of “third-generation biomaterials” development.^36^

## Supplementary Material

Supplemental data

## Abbreviations Used

2D: two-dimensional seeding
DCM: Dichloromethane
HCS: high-content analysis
NSC: neural stem/precursor cells
PLLA: poly(L-lactic acid)
RA: retinoic acid
SD: standard deviation
SEM: scanning electron microscopy
WCA: water contact angle
