# Supplementary material for: In Vitro Testing of Biomaterials for Neural Repair: Focus on Cellular Systems and High-Content Analysis
Source: Biores Open Access. 2016 Aug 1;5(1):201–11. doi: 10.1089/biores.2016.0025 (PMC4991583; doi:10.1089/biores.2016.0025)
Supplement: Supplemental data [file Supp_Data.pdf]

Supplementary Data

Materials and Methods

Cell substrates preparation, characterization and chemical functionalization

To obtain PLLA electrospun scaffolds a 22% w/v polymer solution in 60:40 v/v DCM:DMF was used by means of the following processing conditions: applied voltage = 17kV, grounded drum collector (diameter = 50mm, drum rate = 300rpm for random scaffolds and 6000rpm for aligned scaffolds), needle to collector distance = 20cm, flowrate = 3.0mL/h, , at T = 25°C and relative humidity (RH) = 40-50%. Electrospun scaffolds were kept under vacuum in a desiccator overnight in order to eliminate residual solvents.

Scanning Electron Microscope (SEM) observations of the electrospun scaffolds were carried out using a Philips 515 SEM at an accelerating voltage of 15kV, on samples sputter-coated with gold. The distribution of fiber diameters was determined through the measurement of about 250 fibers by means of an acquisition and image analysis software (EDAX Genesis) and the results were given as the average diameter ± standard deviation.

Static water contact angle (WCA) measurements were performed by means of an optical contact angle and surface tension meter KSV's CAM 100 (KSV, Espoo, Finland), using Milli-Q water. The water drop profile images were collected in a time range of 0–60s, every 1s at RT. Results were averaged on at least five measurements obtained at different areas for each sample.

PLLA electrospun scaffolds and PLLA films were sterilized as follows: incubation 30 min in EtOH 90%, then 30min in EtOH 70%. Ethanol was removed by washing 3x in PBS. After sterilization the scaffolds and the film were incubated in laminin solution 2μg/mL in carbonate buffer 0.1M pH 9.8 for 2h at 37°C and washed 3x with PBS buffer. Cover glass was initially cleaned with a mixture of sulfuric acid (H_2SO_4) and hydrogen peroxide (H_2O_2) (H_2SO_4 :

H_2O_2 3 : 1 v/v) for 30 minutes, washed 3 times with bi-distilled water and treated with laminin as described above for PLLA scaffold. Cultrex® BME was used to coat a cover glass and PLLA electrospun scaffolds and films at 0.25mg/mL concentration for at least 2h. The coating solution was removed just before cell seeding.

Cell cultures.

Human neuroblastoma SH-SY5Y cell line were cultured in DMEM/F12 supplemented with 10% fetal bovine serum, 1x MEM/NEAA, 1x Penicillin-Streptomycin and NaOH 10mM and maintained at 37°C in a humidified 5% CO_2 incubator. RA was dissolved in DMSO, to an intermediate concentration of 16.6mM. SH-SY5Y cells were seeded at a density of $2x10^4$ cells/well in 24 plastic multiwells/dish on different surfaces (BME coated glass, laminin coated glass, laminin coated or not PLLA-FILM, laminin coated or not random and aligned electrospun PLLA scaffold). 24h after seeding SH-SY5Y were treated with RA at final concentration of 10µM and the medium was replaced every three days. To achieve neural differentiation, SH-SY5Y cells was cultured for 7 days.

Cultures of primary cortical rat neurons were obtained from animals within 24h of birth. All the solutions used to prepare the primary neuron cultures were prepared from Krebs buffer (NaCl 1.2M; KCl 0.05M; KH_2PO_4 0.012M; $NaHCO_3$ 0.25M; glucose 0.14M; phenol red 0.1mg/ml). Solution 1 was prepared adding 2.6mg/ml BSA (SIGMA, Saint Louis, MO USA) and $MgSO_4$ 0.03% solution to 9% solution of Krebs buffer in water. Solution 2 was prepared adding trypsin (SIGMA) 0.25mg/ml to solution 1 and, to obtain solution 3, DNase (SIGMA) 0.8mg/ml, SBTI (SIGMA) 0.5mg/ml and 0.04% of $MgSO_4$, were added to solution 1. For solution 4, a solution of 40% solution 3 in solution 1 was prepared and solution 5 was obtained adding $CaCl_2$ 0.0015% to solution 1. All the solutions were filtered using a 0.22µm filter. For the culture medium, Gentamicyn 1% (10mg/ml) (SIGMA), Glutamax 0.25% (GIBCO Invitrogen) and B27 1X (GIBCO Invitrogen) were added to Neurobasal medium (GIBCO Invitrogen, Auckland, NZ USA) and

filtered using a 0.22µm filter. The brain was removed and then handled on a cold surface. The cerebellum and the olfactory bulbs were eliminated, the brain was divided into two hemispheres and the cortex dissected out. Thereafter, the cortex was washed in a Petri plate containing 1ml of solution 1 and then mechanically dissociated using scissors. Tissue was then resuspended in 2ml of solution 1 and centrifuged for 1 minute at 400 x g. The pellet was resuspended in 4.5ml of solution 2, containing trypsin, and incubated for 15 minutes at 37°C under agitation. The trypsined tissue was resuspended with 2ml of solution 4, containing the trypsin inhibitor SBTI, and then centrifuged 1 minute at 400xg. A volume of 0.5ml of solution 3, containing SBTI and DNase, was added to the pellet and carefully resuspended. The suspension was left for 3 minutes at room temperature allowing the deposition of the non-dissociated tissue on the bottom of the tube. The supernatant containing cells was resuspended in 0.5ml of solution 5 and then centrifuged for 5 minutes at 400 x g. The cellular pellet was finally resuspended in 1ml of culture medium and centrifuged 1 minute at 45 x g. The cellular suspension was counted by cell-counter (Scepter™, Millipore, Billerica MA), and seeded at a density of 1 x 10^5cells/cm^2 on 24well plates. Cytosine-Arabinofuranoside (10µM, SIGMA) was added to the culture medium 24h after seeding, in order to inhibit proliferation of the glial cells. To achieve neural differentiation, primary cells were cultured for 15 days.

Neural Stem/Precursor cells (NSCs) were isolated following the Ahlenius and Kokaia protocol with some modifications. Embryos were collected in a 15 ml tube containing ice-cold HBSS (Life Technologies, Milan, Italy). Forebrain were dissected and isolated from embryos and pooled together for a fast wash in HBSS. Tissues were passed in a new 15ml tube containing the culture medium (DMEM/F12 GlutaMAX 1x; 8mM HEPES; 100U/100µg Penicillin/Streptomycin; 0.1x B27 without RA; 1x N-2; 10ng/ml bFGF; 20ng/ml EGF) and mechanically dissected by pipetting using a Pasteur pipette. Cell suspension was than passed in a new 15ml tube leaving in the old tube the undissociated tissue. After cell count, cells were plated at a density of 50cells/µl in a final volume of 3ml in low-attachment 6-well plates (NUNC). Medium was changed every three days, centrifuging the cell suspension at 300 x g for 5 minutes and gently resuspending the cellular pellet

in fresh medium. To obtain secondary neurospheres, cells were centrifuged at 300xg for 5 minutes and incubated in a 0.5 mg/ml trypsin – 0.2 mg/ml EDTA solution in HBSS at 37°C for 15 minutes. After inhibiting trypsinization and subsequent centrifugation, the cellular pellet was resuspended in half fresh/half old medium. Cells were counted and re-plated at the same density. Secondary neurospheres were dissociated and plated at a density of $1x10^4$cells/cm^2 and cells were grown in the same culture medium without mitogens (bFGF and EGF). To achieve full lineage commitment and cell differentiation, seeded cells were cultured for 15 days.

Animal care and treatment were in accordance with the EU Directive 2010/63/EU for animal experiments and in conformity with protocols approved by the Ethical Committee of Animal Experimentation, University of Bologna.

Viability assays

Cell viability was determined by HCS as the percentage of pyknotic nuclei calculated by a manually optimized version Cellomics® Compartmental Analysis BioApplication, using "population characterization". The function allows the identification of a subset of items using Boolean operators, and we identified Pyknotic nuclei as the combination of the smaller and the brighter nuclei. The toxicity study was carried out in compliance with the Good Laboratory Practice guidelines. Thus, we also used the conventional MTT test to compare results obtained from the HCS assay.

MTT assay was performed on primary cortical neurons seeded on 2D cultrex coated surface and semi-3D aligned uncoated scaffolds, at 15 DIV. Cells were incubated in 200µl/well of 0.5mg/ml MTT dissolved in Opti-MEM for 3 hours at 37°C. In order to dissolve formazan crystals, 200µl/well of the solubilization solution containing 80% Isopropanol (IBI Scientific, Peosta, IA, USA), 10% Triton-X 100, 10% HCl 0.1 N (Sigma Aldrich) was added and incubated for 1 hour under gentle shaking at room temperature. Absorbance was measured at 570nm using the

Microplate Reader Model 680 (BioRad, Milan, Italy). Final absorbance values were obtained subtracting mean blank value from the test condition absorbance values.

Immunocytochemistry

Cells were washed in PBS and fixed with 4% paraformaldehyde in 0.1M PBS for 20 min at RT at three different times 3 and 7 Day In Vitro (DIV). In order to minimize non-specific absorption of antibody, fixed cells were incubated in PBS containing 2% of BSA for 1h at RT and washed twice with PBS buffer. The following antisera were used: mouse anti-β-III-tubulin (R&D Systems, 1:2000); rabbit anti-GFAP (Dako, 1:500); rabbit anti-MBP (Dako, 1:250). Subsequently the cells were incubated overnight at +4°C in humid atmosphere with the primary antisera diluted in PBS-Triton 0.1%. After rinsing in PBS (2x10min), cells were incubated with secondary antisera secondary antibody diluted in PBS-Triton 0.1%, for 30min at 37°C. The following antisera were used: anti-rabbit DyLight 568- and anti-mouse AlexaFluor 488-labeled (Invitrogen, 1:500). For nuclear staining, cells were first washed in PBS then incubated 20min with 1μg/ml Hoechst 33258 in PBS-Triton 0.1%. Finally cells were embedded upside-down in a 24 well plate with glycerol, 0.1% 1,4-phenylenediamine (Sigma).

Laminin absorption test.

In order to obtain the mean intensity fluorescence as a function of laminin adsorption into PLLA Scaffold, images of at least three different fields for each sample were taken, furthermore for each of these fields the average intensity of five different areas have been calculated. Each sample was repeated in duplicated and data were expressed as mean intensity ± standard deviation (SD).

Results.

Scaffolds absorption with laminin and Cultrex.

In order to optimize the adsorption of proteins on PLLA scaffolds, two different absorption buffers (PBS 0.1M pH 7.6 or carbonate buffer 0.1M pH 9.8) were tested. Scaffolds were either pre-treated or not with NaOH 0.01N for 5min at RT and incubated with PBS 0.1M pH 7.6 or carbonate buffer 0.1M pH 9.8 laminin solution (2mg/mL) respectively for 2 hours at 37°C. The adsorption of laminin was measured as a function of intensity fluorescence. The results showed that pre-treatment with NaOH did not affect protein adsorption while the incubation with the carbonate buffer increased protein adsorption (n=2; **=$p<0.001$, one-way ANOVA and Tukey post test). This condition was therefore selected for subsequent scaffold preparation *(data not shown).*

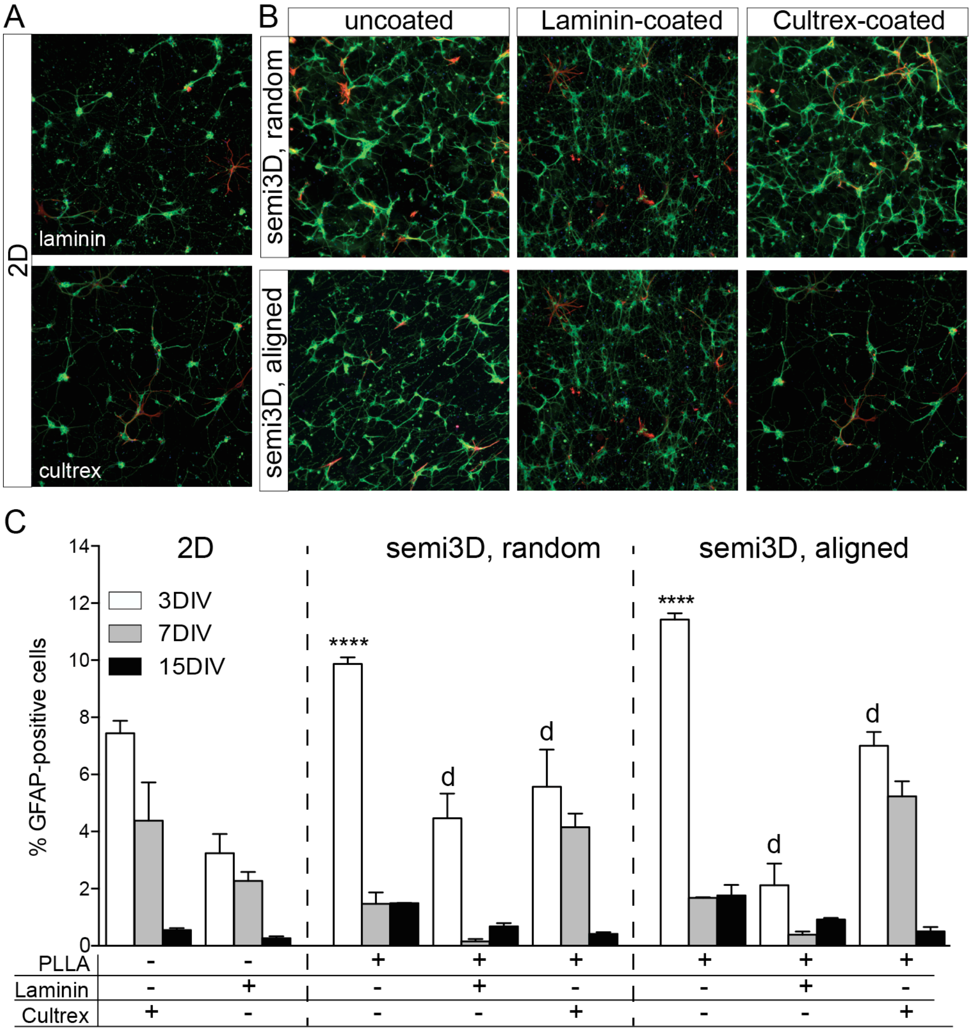

SUPPLEMENTARY FIG. S1. Effect of different scaffolds in inducing GFAP-positive astrocyte lineage in primary cortical neuron cultures. A,B. Representative images of primary cortical neurons at 7 DIV grown on 2D (A) and semi-3D scaffolds (B), stained with Hoechst (blue), β-III-tubulin (green) and GFAP (red). C. The graph represents the GFAP-positive cells percentage measured in Ara-C treated primary cortical neuron cultures.

Data are expressed as mean ± SEM. Statistical analysis: two-way ANOVA ($F_{(14, 48)} = 17.18$) followed by Tukey's post test. Asterisks represent the difference between the semi-3D group vs. the relative 2D control group within the same DIV (****$p<0.0001$), letters indicate the difference between coated semi-3D groups vs. the relative uncoated semi-3D control group (d=$p<0.0001$).

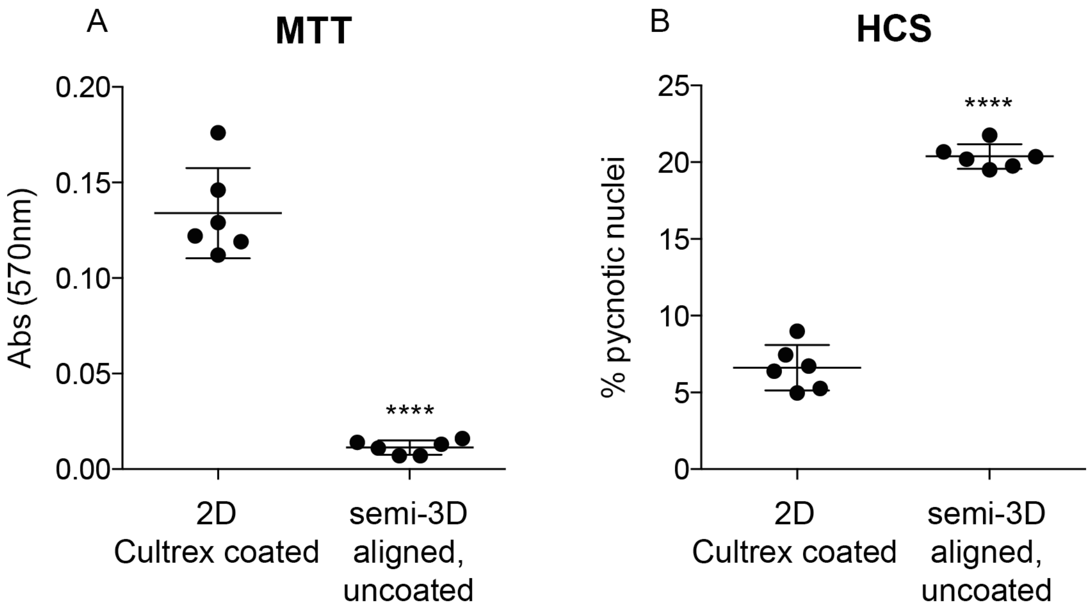

SUPPLEMENTARY FIG. S2. Cell viability on PLLA electrospun fibers (2D Cultrex coating vs semi 3D aligned uncoated fibres) evaluated by a conventional viability test based on mitochondria function (MTT) (A) by HCS (nuclear pyknosis) (B). Bars represent mean ± SD of six independent wells from a single experiment (coefficient of variation: 2D cultrex coated, MTT = 17.62% HCS = 22.35%; semi-3D aligned uncoated, MTT = 32.86% HCS = 3.90%) Statistical analysis: Student's t test, ****$p<0.0001$.
